# Health Literacy–Related Safety Events: A Qualitative Study of Health Literacy Failures in Patient Safety Events

**DOI:** 10.1097/pq9.0000000000000425

**Published:** 2021-06-23

**Authors:** Andrea K. Morrison, Cori Gibson, Clarerita Higgins, Michael Gutzeit

**Affiliations:** From the * Section of Emergency Medicine, Department of Pediatrics, Medical College of Wisconsin, Milwaukee, Wis.; † Children’s Wisconsin Quality Department, Milwaukee, Wis.; ‡ Children’s Wisconsin, Chief Medical Officer, Milwaukee, Wis.

## Abstract

**Introduction:**

Communication failures are the leading root cause of safety events. Although much communication research focuses on the healthcare team, there is little focus on communication with patients and families. It is not known what deficits in health literate patient communication lead to patient safety events. We aimed to identify themes of health literacy–related safety events to describe the impact of health literate communication on patient safety.

**Methods:**

The safety events were entered into a system-wide self-reported safety event collection database. A patient safety specialist trained in health literacy prospectively tagged events for health literacy. The authors retrospectively queried the database for all health literacy tagged events during 9 months (September 2017–May 2018). The authors reviewed and independently coded health literacy-associated safety events. Qualitative content analysis of events facilitated by software (NVivo) was completed to identify the health literacy–related safety event themes.

**Results:**

Health literacy events comprised 4% (152/3911) of self-reported safety events during the 9 months. Main themes of the health literacy safety events related to (1) medication; (2) system processes; and (3) discharge/transition. Subthemes of each of the events further described the event types. Health literacy–associated safety events encompass all safety event outcomes (near miss, precursor, and serious safety events).

**Conclusions:**

Health literacy–related safety events occur in the healthcare environment. This review characterizing health literacy–related safety events prioritizes areas to implement health literate safety practices. Many opportunities exist to address communication-related safety events around medication, system processes, and discharge using health literate best practices.

## INTRODUCTION

Clear, effective communication is essential to ensure patients and families understand health information and care instructions.^1^ Deficiency in clear communication leads to poor outcomes and patient and family experience.^2^ Despite the importance of clear communication, the study and literature surrounding communication in safety focuses on the communication between the healthcare team, with few studies focusing on the miscommunications between families and the healthcare team.^3^ Reviewing and analyzing communication breakdown related to safety events allows the opportunity to learn and identify safety improvements.^4^ One approach to evaluating communication between the healthcare team and patients’ families includes using a health literacy focus.^5^

Over 90 million American adults have low health literacy impacting their ability to understand health information to make health decisions.^6^ Health literacy goes beyond merely the ability to read. It also includes processing, making decisions about health behaviors, and acting on health information.^7^ The healthcare system’s ability to communicate with patients and families describes the organization’s health literacy.^5,8^ Improving organizational health literacy is the critical area in which the healthcare field can ensure patient and family understanding.

Suboptimal or inconsistent communication is one of the top 3 root causes of sentinel safety events.^9^ Although becoming a health literate organization has gained importance, most organizational safety departments have not adopted health literacy to improve the communication gap leading to harm. As an example of how organizations can improve, our organization’s Health Literacy Task Force proposed an implementation strategy to improve health literacy using the “Ten Attributes of a Health Literate Health Care Organization” as drivers of improvement (Table 1).^5^ The task force conducted a practice assessment to identify needs and engaged leadership through speaking at various forums.

**Table 1.** Ten Attributes of a Health Literate Organization^5^

	Attribute Description
1	Has leadership that makes health literacy integral to its mission, structure, and operations.
2	Integrates health literacy into planning, evaluation measures, patient safety, and quality improvement.
3	Prepares the workforce to be health literate and monitors progress.
4	Includes populations served in the design, implementation, and evaluation of health information and services.
5	Meets needs of populations with a range of health literacy skills while avoiding stigmatization.
6	Uses health literacy strategies in interpersonal communications and confirms understanding at all points of contact.
7	Provides easy access to health information and serves and navigation assistance.
8	Designs and distributes print, audiovisual, and social media content that is easy to understand and act on.
9	Addresses health literacy in high-risk situations, including care transitions and communications about medicines.
10	Communicates clearly what health plans cover and what individuals will have to pay for services.

Reproduced with permission from the National Academy of Sciences, Courtesy of the National Academies Press, Washington, D.C.

Few studies tie safety events to a need for improving health literacy practices in an organization.^10^ Through this study, we aimed to identify themes of health literacy-related safety events. By identifying these themes, we will (1) describe the impact of health literate communication on patient safety and (2) aid organizations in prioritizing health literate safety practices.

## METHODS

The tertiary Midwest Children’s hospital has nearly 70,000 emergency department visits per year, 298 beds, and over 70,000 inpatient days per year. Front line staff enter safety events into a system-wide self-report safety event database, MIDAS+ Incident Entry System (Conduent Inc., Florham Park, N.J.). Staff entering the event can choose an event type from a list (eg, medication-related, IV medications, laboratory, and drains/lines/tubes). The event collection includes location, role, and a typed narrative. Care areas respond to events with a narrative about a further investigation into causes and improvements required. A safety specialist then reviews events for actionable items and codes event types into predetermined categories (central line, delay, equipment, IV blood, etc.). The Health Literacy Task Force recommended adding a health literacy event type to the database to aid in characterization. The Health Literacy Task Force comprises individuals from multiple areas of the health system interested in improving the organization’s health literacy focus. One safety specialist (C.H.) and member of the task force reviewed all safety events as a part of regular duties and coded for health literacy after understanding the event as a part of a regular investigation. The health literacy event definition included events in which the organization did not make it easy for people to navigate, understand, and use information and services to take care of their health, drawing from health literacy and health literate organization definitions.^5,7^ Failure to follow the “Ten Attributes of a Health Literate Organization” was considered in evaluating events for inclusion (Table 1).^5^ The safety event database was retrospectively queried for all health literacy tagged events during 9 months from September 2017 to May 2018. Any identifying characteristics (names and dates) were redacted from the narrative transcripts by the safety specialist.

The authors (A.K.M., C.G., and C.H.) independently evaluated and coded the safety event narratives. They reviewed the entire narrative, including responses from care areas. They also coded the healthcare area, roles involved, and safety event types. During group meetings, the authors discussed all events and agreed on the inclusion and final coding of an event. Disagreements were resolved by consensus. Novel themes emerged as coding progressed, and the theme categories were created using inductive reasoning. Previous events were reviewed for inclusion in the new category. After reviewing 9 months of data, no new themes emerged for the last month of data, and the authors determined thematic saturation was reached.

The study team developed a tentative set of themes from the literature about health literacy safety issues.^11^ The authors used content analysis to categorize the events based on health literacy–related characteristics and discussed events that did not fit in previous categories. A new theme or subtheme was added if multiple events existed, and a consensus was reached to add another theme category. NVivo 11.0 (QSR International, Burlington, Mass.) aided coding and analysis.

Due to concerns in reviewing events from a protected environment, only illustrative cases with themes and subthemes are presented for results.

## RESULTS

In the 9 months, there were 156 events labeled as health literacy–related safety events. The authors agreed that 4 events did not fit the criteria for a health literacy-related event and excluded them from the analysis. The study included 152 (4%) events out of 3,911 total safety events. Health literacy–related safety events were reported an average of 4 times per week. The events mainly involved inpatients (59%), but also outpatients (primary care and specialty care) (29%), and emergency department patients (8%). The distribution for health literacy safety events’ location resembles the overall safety event distribution (71% inpatient, 19% outpatient, and 8% emergency department). The majority of events were precursor events (91%), events that reached the patient, and minimal or no detectable harm. But there were also near-miss events, events that did not reach the patient (7%), and few serious safety events (2%) that reached the patient and resulted in moderate-to-severe harm. Serious safety events included misunderstanding of equipment resulting in severe, temporary harm, and 2 instances of medication dosing misunderstanding resulting in moderate temporary harm requiring rehospitalization.

The main themes of the safety events related to (1) medication (53%); (2) health system processes (27%); and (3) discharge/transition (20%). The themes will be described further with subthemes in the following sections with illustrative cases in Table 2.

**Table 2.** Health Literacy–Related Event Themes and Illustrative Cases

Event Themes	Illustrative Cases
Medication (Health System Entry, Encounter, Transition, and Self-management)	
Mistakes on admission medication reconciliation	Admitted patient received double medication dose for over 1 wk while inpatient due to unclear medication history taking on admission.
Unclear discharge medication instructions	Seizure medication increase after hospitalization planned over several weeks. Parent returned to home dosing according to the prescription bottle. Parent not given clear schedule for increasing dose.
Multiple conflicting instructions	Prescription medication bottle and discharge papers gave dosage information. Other documentation (chemotherapy roadmap) parent given recommended different days for medication administration.
Clinic administered medications	To identify patient, staff confirmed vaccines to be given, rather than patient name and birthdate, with father before administering vaccines. Patient received sibling’s vaccines.
System processes	
Unclear navigation assistance	Staff was unclear about keeping blood bands on after blood draw. Patient’s procedure delayed due to blood bands not on patient.
Relying on parents	After MRI, staff asked parents if the child had a programmable ventricular shunt. Shunt was not reprogrammed and did need to be. Child returned to ED with headaches.
Failure to address language barrier	Infant with UTI given prescription for once a day antibiotics. Unclear discharge instructions led to twice daily dosing. Antibiotics course finished too early. Patient back to ED with pyelonephritis and was admitted.
Discharge/transition (nonmedication)	
Unclear written information	Discharge documentation recommended bolus feeding, but patient has nasojejunal tube and can only receive continuous feeds.
Unclear verbal teaching about after visit needs	Parent did not receive clear information for how to collect or prepare for labs to be done after hospitalization. Led to delay in collecting laboratories.
Unclear equipment information	Patient with new g-tube. Unclear teaching led parents to not understand how to change the tube when it fell out, requiring visit to ED. Patient taught how to use knee braces incorrectly and fell due to error. Unclear instructions on how to obtain initial equipment and replacement equipment.

ED, emergency department; MRI, magnetic resonance imaging; UTI, urinary tract infection.

### Medication Health Literacy–Related Safety Events

The following sections describe how the lack of clarity in medication plans led to difficulty understanding the medication plan, contributing to safety events.

#### Mistakes on Admission Medication Reconciliation

Mistakes made on admission medication reconciliation occurred mainly due to unclear medication history taking. The errors made included incorrect medication reconciliation, failure to confirm dosages, carrying forward the dose in the medical record, and failure to obtain home dose from alternative sources when medication information was not known. The medication errors were made both with errors in medication dose and dosing interval. Often staff identified these errors at the time of discharge, and the error had been conveyed throughout the hospitalization, in some cases over a week.

#### Unclear Written Discharge Medication Instructions

Communication errors with medication happened at the time of discharge. These included written discharge materials with unclear medication dose, unclear dosing interval, and missing medications. The unclear medication dose often occurred with medications that are difficult to put in the electronic medical record (EMR) due to ranges (eg, insulin). Unclear doses also occurred with changing schedules (eg, narcotic or steroid medication wean, or antiepileptic medication wean or increase). Errors in the dosing interval occurred with variable medication schedules (eg, chemotherapy and azithromycin) and requests to increase medication by intervals (eg, antiepileptic medication).

Other unclear information on written discharge instructions included the incorrect route of administration. Typically, the errors in route resulted from mistaken instructions to administer by mouth rather than by g-tube. Still, there were other examples of the unintended ordering of intravenous or intramuscular routes. Additionally, unclear medication plans occurred regarding the continuation of a dose at home after a dose was given during care (eg, steroid given in the ED) and which pharmacy to retrieve medications from (eg, medications required dispensing from a specific pharmacy).

#### Multiple Conflicting Instructions

Communication errors occurred with multiple conflicting instructions. The conflicting information included written versus verbal communication about dose or dosing interval. Conflicting instructions also occurred with multiple written instructions. Examples of conflicting instructions include the after-visit summary compared to pharmacy labeling, chemotherapy “roadmap” compared to pharmacy labeling or after-visit summary, conflicting information within the after-visit summary, or parent given multiple dosing strategies.

#### Clinic Administered Medications (Immunizations)

The events surrounding immunizations were missing clear communication about vaccines and clinic procedures for vaccines. For example, the clinic staff used jargon to describe the clinic procedure, which led to the error. In other cases, unclear communication around informed consent and vaccine administration took place. The vaccine administrator did not identify the correct patient, did not give the correct time, or did not communicate what immunization was being given.

### System Processes Health Literacy–Related Safety Events

As outlined in the following sections, unclear communication in a health system can lead to difficulty understanding health system processes and contributes to safety events and poor experience outcomes.

#### Unclear Navigation Assistance

Patients and families need assistance with navigating the health system to access the needed services and care. Safety errors occurred when staff gave unclear information about hospital or clinic procedures, home care, pharmacy system, or after visit procedures. In these circumstances, families did not understand how to obtain the needed services, equipment, or medication (ie, gave home medications in the hospital, could not obtain equipment, did not understand how to obtain medication refills).

#### Relying on Parents for Complex Health Information

Safety events occurred when staff relied on parents for specific complex health information. These safety events occurred around immunizations, requiring parents to recall precise medical information (ie, ventricular shunt type), or when teams used the parents to hand off information between teams. In all of these instances, other information systems were available to verify a patient or device.

#### Failure to Address Language Barriers

Several safety events occurred because of the failure to address language barriers. Safety events occurred when clinicians relayed verbal information without an interpreter present and gave written information without translation. Errors occurred when staff gave information about a medical problem, navigation, discharge instructions, or medication.

### Discharge/Transition Health Literacy–Related Safety Events

As illustrated in the sections below, the lack of clarity in discharge led to difficulty understanding treatment plans and contributed to safety events.

#### Unclear Written Discharge Information

Similar to the medication events, discharge events included unclear written discharge information. The information in these cases was missing or incorrect. Nearly half of this event type consisted of unclear information about the feeding plan (schedule, type of formula). Other missing or incorrect information included appointments, contacts (physicians and home care agency), call parameters, and care of the disease (ie, wound care and postoperative care).

#### Unclear Verbal Teaching about after Visit Needs

Verbal teaching at the time of discharge or transition contained conflicting or incorrect information about follow-up appointments or verbal instructions as compared to written information. There was also a lack of follow-up information, including appointments, reasons to follow-up, and call parameters. A prominent area of unclear verbal teaching at discharge included preparation for a follow-up appointment. This unclear information included obtaining labs or conducting lab collection at home properly (ie, stool studies), injection plans, planning for the future procedure, and the expected course after the visit.

#### Unclear Equipment Education

Education about equipment at the time of discharge/transition was unclear, leading to home equipment issues. The equipment education was unclear about how to use the equipment or troubleshoot problems after leaving care. Patients and families did not receive clear education about anticipating future equipment needs, plans for future equipment needs, or anticipating future maintenance issues.

## DISCUSSION

We identified suboptimal communication themes around medication, health system processes, and discharge/transition occurring 4 times per week through a review of health literacy–related safety events. Identifying these error themes and subthemes importantly describe errors that keep our health system, and other similar health systems, from being health literate healthcare organizations. To our knowledge, this review is the first of its kind and allows further understanding of safety events occurring related to health literacy.

Health literacy–related communication errors lead to adverse outcomes such as medication administration errors, delays in treatments and procedures, understanding of one’s medical condition, and adherence to treatment plans.^11–13^ Our results confirm failure to consistently use health literacy strategies when communicating with patients and families leads to safety errors.^10^ Numerous studies demonstrate the connection between health literacy and the higher risk for medication errors, including poor understanding of medication names, indications, instructions and adherence, and medication administration.^14,15^ Like our findings, others have also found that parents frequently struggle to understand and execute discharge instructions, often lacking essential information, leading to errors.^16^ Despite best intentions, limitations in medication entry, and visual display of medication information on written instructions using an EMR may lead to confusion, especially with complex medication plans and variable dosing schedules.^17^

Children and families face many health literacy–related challenges related to health system processes, including participation in hospital procedures, understanding care plans, and preparing for discharge.^18^ Safety events occurred had a lack of understanding of system procedures. Providers’ use of medical jargon and lengthy, complex questions when obtaining a history creates difficulty for some families to provide a complete and accurate picture.^19,20^ This difficulty is exacerbated in parents with children with chronic medical conditions, or those whom English is not a primary language, who may struggle with understanding their child’s condition.^11^ Failure to address language barriers through the use of qualified interpreters and translated written materials leads to incomplete assessments and misunderstandings about care plans, compromising patient safety.^21^

Verbal and written communication at the time of discharge or transition is complex. As we found, written discharge instructions often contain too much information and are confusing. This confusion creates parental misunderstanding resulting in errors.^16^ Patients and families have expressed the need for more clarity in discharge instructions and awareness of outpatient resources.^22^ Parents desire clear information regarding medical equipment use, practice and troubleshooting issues to help them feel prepared, and an understanding of how to mitigate any issues that may arise in the home.^23,24^

Communication errors between the healthcare team, patients, and families are common but understudied in patient safety.^3,25^ Much of the research focuses on the communication between members of the healthcare team. By including healthcare team interprofessional communication along with patient and family communication, it is difficult to discern the details or adopt safety techniques for communication errors with patients and families. Many safety techniques adopted for communication focus on the healthcare team and neglect the communication with patients and families. One tool, acknowledge-introduce-duration-explain-thank, focuses on communication with patients and family.^26^ The technique recommends speaking clearly with the family to promote safety without specific methods of clear communication such as plain language. Health literacy techniques in communication can fill that gap in how to communicate clearly. In practice, implementing specific health literacy techniques for communication during inpatient rounds effectively improved patient safety.^3^

Despite the demonstrated safety ramifications, few organizations are currently responding to health literacy as a safety issue. Health systems could follow the “Ten Attributes of a Health Literate Organization” to protect patients from harm.^5^ Health systems should use this event review’s themes to conduct a needs assessment and build future interventions. An organizational assessment to identify health literacy–related factors in one’s organization can serve as a starting point to help identify gaps.^27^ Organizations could track and analyze health literacy–related safety events to allow for organizational learning and needs assessment. Encouraging additional detailed reporting regarding communication errors may help identify future improvement opportunities.^4^

System changes prioritizing improvements focused on the “Ten Attributes of a Health Literate Organization” are needed (Table 1).^5^ Communication between the healthcare team, patients, and families has been identified as a high safety priority topic by pediatric clinicians, healthcare leaders, and families.^28,29^ Developing policies and standards around clear and effective communication in healthcare settings have been recommended.^30^ Health literacy strategies to improve spoken and written communication and tactics to address health literacy challenges specific to the inpatient hospital setting will prove useful.^3,11,18^ Additional evidence-based recommendations to improve communication between healthcare providers and patients and families are available and can be used to address identified gaps.^29,31^

Moving forward at our organization, our Health Literacy Task Force will use the themes and subthemes generated in this study to prioritize our work. The themes focused our efforts on discharge medication teaching at discharge and immunization safety, aligning with institutional priorities. Our team drafted performance standards for immunization and medication communication for use in improvement efforts. Our team will continue to educate systematically about health literacy, including all new hires. Future discharge medication communication initiatives to improve discharge teaching and parental dosing accuracy include a standard discharge bundle and high-intensity discharge bundle, stratified through risk-factors, including health literacy.^32^

### Limitations

In this retrospective review, self-report limits the findings. Many safety events occur and are never reported; some occur after discharge without capture in the safety event reporting system. The institutional reporting of safety events has not focused on communication-related events, and likely communication-related events are underrepresented in this study. Additionally, not all safety events were reviewed by the entire research team for inclusion. The single reviewer may have missed some events.

Parents’ or patients’ voices of safety are not captured within the safety reporting infrastructure unless the narrative contains information about the experience. Parent and patient experience of safety practices could expand or change the themes of this review.

The authors established a possible path of how a lack of health-literate practices can lead to safety events; however, this study’s qualitative nature does not establish causality.

## CONCLUDING SUMMARY

A lack of health literacy-focused practices impacts patient safety in understanding medication, health system processes, and discharge/transition. Organizations need to review and address health literacy–related safety events to prevent future harm. There are many opportunities to address communication-related safety events using health literacy best practices.

## DISCLOSURE

The authors have no financial interest to declare in relation to the content of this article.
